# Cone-Beam Computed Tomography for the Evaluation of Dental Pulp Chamber Volume: Implications for Clinics and Teaching

**DOI:** 10.3390/dj12040095

**Published:** 2024-04-03

**Authors:** Maria Llacer-Martínez, Benjamín Martín-Biedma, María T. Sanz, Juan I. Aura-Tormos, Pablo Fos-Galve, Zulima Fernández-Muñiz, José A. Vega, Mar Jovani-Sancho

**Affiliations:** 1 Departamento de Odontología, Facultad de Ciencias de la Salud, Universidad Cardenal Herrera-CEU, 46115 Valencia, Spain; mllacermartinez28@gmail.com (M.L.-M.); juan.aura@uv.es (J.I.A.-T.); dr.pablofos@gmail.com (P.F.-G.); 2 Departamento de Cirugía y Especialidades Médico-Quirúrgicas, Universidad de Santiago de Compostela, 15705 Santiago de Compostela, Spain; benjamin.martin@usc.es; 3 Departamento de Didáctica Matemática, Universidad de Valencia, 46010 Valencia, Spain; m.teresa.sanz@uv.es; 4 Departamento de Matemáticas, Universidad de Oviedo, 33003 Oviedo, Spain; zulima@uniovi.es; 5 Departamento de Morfología y Biología Celular, Universidad de Oviedo, 33003 Oviedo, Spain; 6 Facultad de Ciencias de la Salud, Universidad Autónoma de Chile, Santiago de Chile 7500912, Chile

**Keywords:** cone-beam computed tomography, computed micro-tomography, dental pulp chamber volume

## Abstract

The dental pulp chamber volume is a fundamental measurement in the field of endodontics, but also in forensic sciences, teaching and training, or tissue engineering. This study evaluates the precision of cone-beam computed tomography (CBCT) in comparison with computed micro-tomography (micro-CT) in evaluating the pulp chamber volume of the upper central incisors ex vivo. The intra-operator and inter-operator errors were evaluated, and the results for the two techniques were compared with those of a T-test for paired samples. The intra-operator and inter-operator errors were >0.05, indicating adequate reproducibility in each operator and no significant differences between their measurements. On the other hand, no significant differences between the two measurement techniques were found. The present results demonstrate that CBCT is a precise, feasible, and reproducible technique for the evaluation of the dental pulp chamber volume ex vivo. The results provided with this method are useful for different medical domains but also for the teaching and training of undergraduate and postgraduate students. Furthermore, the findings of this study carry significant clinical implications, as the accurate assessment of the pulp chamber volume is critical in the diagnosis and treatment of various endodontic conditions. The ability of CBCT to provide reliable 3D dental anatomy measurements can enhance the planning of endodontic treatments by allowing for a better understanding of the internal tooth morphology. Additionally, the precision and reproducibility of CBCT in assessing the pulp chamber volume can contribute to improved clinical outcomes and reduced complications during endodontic procedures. These findings further support the increasingly vital role of CBCT in modern clinical practice and underscore its value as an indispensable tool in the field of dentistry.

## 1. Introduction

Determining the dental pulp chamber anatomy and volume is an important goal in dentistry, as they provide information that contributes to correct diagnosis, treatment planning for several dental pathologies, and the monitoring of cases over time [1]. In this context, computed micro-tomography (micro-CT) is currently regarded as the gold standard technique to study the root canal anatomy and apical foramens, evaluate the volumetric pulp space, and perform follow-up interventions [2,3,4,5]. However, several factors limit the use of micro-CT in daily dentistry practice. They include the high prices of the device and equipment as well as the data analysis software. Moreover, the use of micro-CT is not possible in medical practice because of the long time needed for scanning and digital reconstruction. Moreover, due to volume limitations, micro-CT does not allow one to scan the full head of a living person but is restricted to extracted teeth or jaw segments containing teeth, and any case is only used in studies ex vivo [3,6,7].

Recently, it has been reported that small-field cone-beam computed tomography (CBCT) allows one to obtain good-quality three-dimensional tooth images at low levels of radiation, without superimposing the neighboring anatomical structures. CBCT has been incorporated into clinical practice in a variety of disciplines, including periodontics, oral and maxillofacial surgery, implantology, and forensic dentistry [7,8,9]. Importantly, in endodontics, it has been used to identify the number of root canals with great accuracy [10], and its use is particularly relevant in the diagnosis of dental fractures and resorption [6]. In fact, the numbers of images captured by CBCT now surpass those of classical digital radiology in dentistry diagnosis and treatment planning [7]. Although the images obtained with CBCT would appear to offer high precision in all spatial planes, assessments of their reliability and accuracy have been rare [3]. The variables studied have included measurements of the areas and diameters of the root canals [6], linear measurements on digital models [11], linear measurements in the field of implant dentistry [8], calculations of dental volumes [1], and the assessment of volumetric distortion artifacts in endodontically treated teeth [12]. CBCT is also used to perform measurements of pulp chamber volumes and to estimate age. In fact, dental age estimation in living individuals as well as in cadavers is one of the most frequent tasks undertaken by forensic odontologists. It is well known that ageing is accompanied by the formation of secondary dentine and a consistent reduction in the tubular lumen diameter, leading to a reduction in the volume of the pulp chamber [13,14,15,16,17]. Indeed, measurements of the pulp chamber are necessary in preparing 3D models to be used as scaffolds for in vitro pulp reconstruction [18,19,20]. Importantly, CBCT is also a good tool to create realistic tooth models for preclinical teaching and training to endodontic postgraduates [21,22,23,24]. Nevertheless, although CBCT images have a high precision in all spatial planes, studies analyzing their reliability and accuracy are scarce; moreover, when used to evaluate the dental pulp chamber, the underestimation or overestimation of measurements has been identified [8,12,25].

In the last few years, studies have reported the use of CBCT to determine the volume of the dental pulp chamber, but, to our knowledge, studies corroborating its accuracy and reproducibility are not available. Thus, this study was designed to validate the precision of CBCT by comparing pulp chamber volume measurements ex vivo obtained with the Promax^®^ 3D Max CBCT (Planmeca Inc, Roselle, IL, USA) and those obtained with micro-CT. A recent study by Maddalone et al. [26] concluded that CBCT is suitable for pulp chamber morphology evaluation, with limitations in detecting the anatomical variability of small branches in the root canal system.

The present study aimed to assess the dimensions of the pulp chamber of the upper central incisors using CBTC images from ex vivo teeth, evaluating the precision and reproducibility of this method in assessing the pulp chamber volume. The hypothesis is that CBCT can provide accurate and reproducible measurements of the dental pulp chamber volume, which is crucial for correct diagnosis and treatment planning in dentistry.

## 2. Materials and Methods

### 2.1. Sample Selection

Thirty upper central incisors were used in this study. The sample size was based on previous studies by Domark et al. [7], which used 27 molars; Grande et al. [27], who studied 30 premolars using CBCT and micro-CT; and more recent studies like that of Puleio et al. [28], where 10 single-rooted teeth were used.

The material was collected from the Dental Clinic of the Universidad CEU Cardenal Herrera in Valencia (Spain) and was extracted for periodontal reasons. Patients’ informed consent was obtained prior to sample collection. The pieces showed closed apices and no major destructions. Teeth associated with an anatomical abnormality, internal or external resorption, prosthetic restoration, orthodontics, or endodontic treatment were excluded [11,29]. After extraction, the pieces were disinfected with 5% sodium hypochlorite for 2 h, and then stored at 4 °C in distilled water until use. The study was approved by the Ethical Committee at Cardenal Herrera University (Valencia, Spain; Reg. No. CEI19/089).

### 2.2. CBCT Study

For image acquisition, the teeth were placed on a silicone impression putty base (Express 2 Putty Quick, 3M ESPE) in groups of ten. A mark was made to place them in a position to mimic an arch and they were scanned using the Promax^®^ 3D Max CBCT unit (Planmeca Inc, Roselle, IL, USA). The images generated were captured by applying the following parameters: a field of view of 8 × 5 cm, 120 KV, 8 mA, and a voxel size of 0.2 mm, according to [30]. The imaging time was 8.03 s, and data were reconstructed at a slice interval of 1 mm. The measurement of the pulp volume in CBCT was obtained by selecting the cube tool for automatic volume measurement, using the grayscale threshold. In each tooth, an initial point in the pulp was selected from which, using the 3D region growing tool with the preset ‘root cavity’, a 3D model was generated, and the pulp volume in cm^3^ was obtained.

CBCT images were stored in Digital Imaging and Communication in Medicine (DICOM) format. Then, two researchers (dentists with over 10 years of experience) independently took measurements of the pulp chamber volume using the Planmeca Romexis software (version 5.3.4.39).

**Figure 1:**
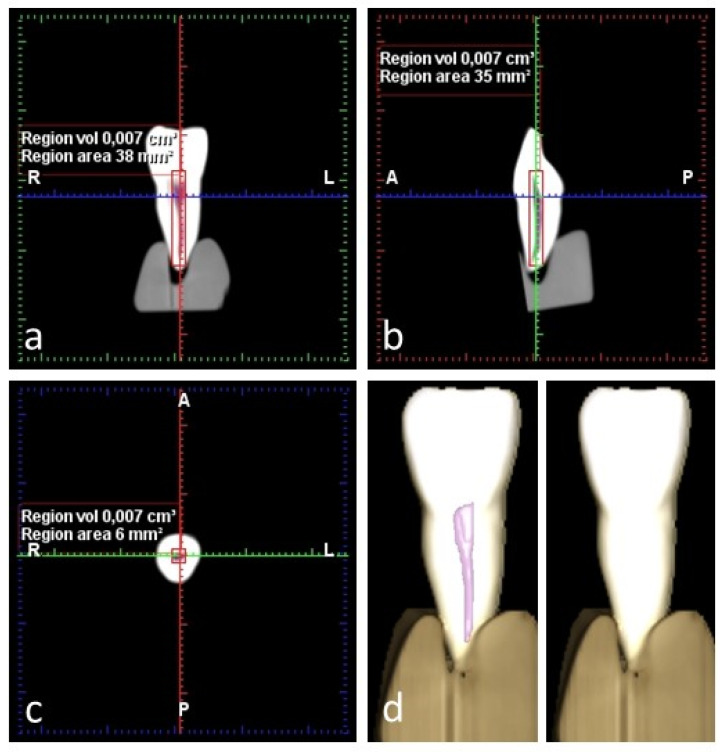
CBCT image of a specimen showing pulp volume measurement in three planes: (**a**) coronal plane; (**b**) sagittal plane; (**c**) axial plane; (**d**) 3D reproduction of pulp volume. R, L, A, P stands for Right, Left, Anterior, Posterior.

The procedure was conducted in a double-blind manner and carried out twice, separated by an interval of 15 days. The intra- and inter-examiner errors were calculated since pulp chamber volume measurement is characterized by an element of subjectivity, as the operator must select the area to be measured. The pulp camber volume was obtained using the grid tool to measure volumes automatically using the grayscale threshold, as in previous studies [31]. For each tooth, a start point was selected from which the 3D region growing tool was set to the option ‘root cavity’. A 3D model was generated and the pulp volume measured in cm^3^ (Figure 1).

### 2.3. Micro-CT Study

**Figure 2:**
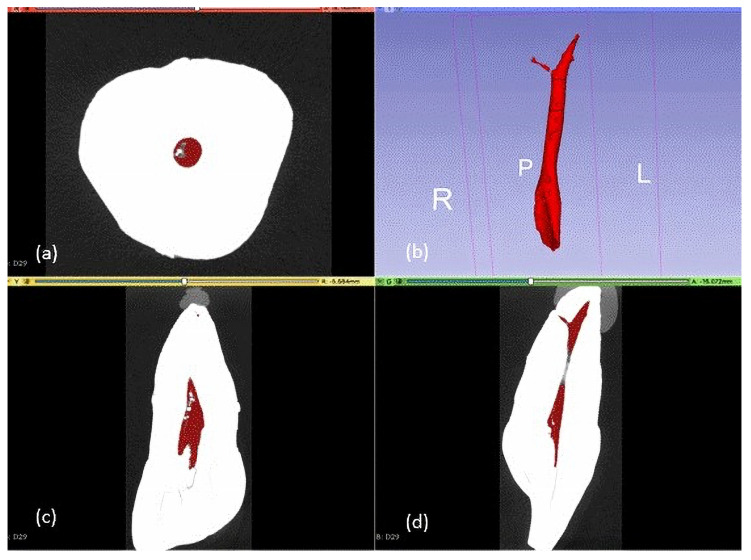
Micro-CT image of a specimen showing pulp volume measurement in three planes: (**a**) axial plane; (**c**) coronal plane; (**d**) sagittal plane; (**b**) 3D reconstruction of pulp volume, where R, P, L, stands for Right, Pulp, Left.

The same pieces were then analyzed with micro-CT. The study was carried out at Centro Nacional de Investigación sobre la Evolución Humana (CENIEH; National Center for Human Evolution) Burgos, Spain, using a micro-CT instrument (Phoenix VltomelXs240, GE Sensing & Inspection Technologies Phoenix X Ray, Wunstorf, Germany). The scanning conditions were set at 120 kV and 120 µA, with a 0.2 mm Cu filter, 19 µm voxel size, and 0.2 step rotation. To minimize ring artifacts, air calibration of the detector was performed before scanning. Each sample was rotated 360° within an integration time of 2 s. The mean scanning time was approximately 1 h. Thereafter, data were exported in DICOM format and pulp volumes were calculated with the 3D Slicer software (version 4.10.2). Segmentation was performed using the grayscale threshold tool, differentiating coronal dentin from the root cementum. An examiner trained in the software was blinded for segmentation, evaluating the three planes simultaneously (axial, sagittal, and coronal), eliminating all calcifications found throughout the pulp from the coronal to apical areas. The process took between 45 min and 3 h per tooth. Then, 3D models were generated, measuring the pulp volume in mm^3^ automatically. Phoenix Datos/x 2 reconstruction and the 3D Slicer software were used for root canal visualization and reconstruction. For reconstruction, a median filter was applied, and the beam-hardening correction was set at 80%. Contrast limits were applied automatically following the GE micro-CT manufacturer’s instructions. The 3D Slicer software was used to visualize the 3D volumes and to measure the root canal volume (Figure 2).

### 2.4. Statistical Analysis

The consistency and reliability of the measurements was evaluated by the two investigating dentists to reduce the bias and improve the validity of the results.

Firstly, the mean (average) of the measurements taken by two different operators was calculated to reduce the potential for bias that might be associated with individual operator measurements. The Statistical Package for the Social Sciences (SPSS) version 22.0 for Windows (IBM, Chicago, IL, USA) was used for the analysis, and the descriptive analyses involved calculating the means and standard deviations of the measurements.

The normality of distribution was assessed for both micro-CT and CBCT volume measurement using the Kolmogorov–Smirnov (KS) test. This is a type of statistical test used to assess whether a dataset follows a specific probability distribution, such as a normal or Gaussian distribution. The test compares the empirical distribution of the observed data with the theoretical distribution expected under the null hypothesis that the data follow the specified distribution. If the *p*-value associated with the test is greater than a predefined threshold (commonly 0.05), there is not enough evidence to reject the null hypothesis, suggesting that the data may follow the specified distribution.

The test compares the empirical cumulative distribution of the data with the expected cumulative distribution under the null hypothesis. The *p*-values reported for both tests were 0.20, suggesting that the data did not deviate significantly from a normal distribution, i.e., they did not provide sufficient evidence to reject the null hypothesis at the 0.05 significance level (a commonly used threshold). The significance level represents the probability of making a type I error or incorrectly rejecting a true null hypothesis. A lower significance level reduces the probability of making type I error but may increase the probability of making type II error, or failing to reject a false null hypothesis. Therefore, the significance level should consider the nature of the problem, the practical implications, and the risk tolerance for statistical errors.

The KS test is used as a goodness-of-fit test, assessing the extent to which the sample data fit a specified theoretical distribution. It is also particularly effective in detecting differences between the observed data and the expected distribution in the tails of the dataset, providing a comprehensive assessment of the entire distribution.

Student’s *t*-test was performed to compare the means of the two groups. In the context of paired data, such as data before and after a treatment or data from two matched groups, like those obtained by two different operators, the *t*-test is used to determine if there is a significant difference between the means of the two groups or one that is simply the result of chance. The *p*-value associated with the test indicates the probability of obtaining results as extreme as those observed if the true difference between the group means is zero. If the *p*-value is less than a predefined threshold (usually 0.05), there is a statistically significant difference between the means of the two groups.

This comparation was possible because the data followed a normal distribution and the variances of the two groups were approximately equal (homoscedasticity). The *p*-value associated with Fisher’s test was 0.586, suggesting that there was no significant evidence to reject the null hypothesis of equal variance. In addition, the paired samples *t*-test was performed to compare the means of the two related datasets and to assess whether there was a significant difference between the two measurement conditions, assuming the normality of the differences and homogeneity of the variances. A significance level for the *t*-test of 0.05 was chosen.

## 3. Results

The pulp volume of the 30 teeth ex vivo was obtained using CBCT and micro-CT images. Next, the measurements were compared to verify whether CBCT was an accurate tool in determining the dental pulp volume.

As during the procedure, the operator had to select the area to be measured and this leads to an element of subjectivity; the intra- and inter-examiner errors were calculated.

The intra-operator error calculated reached a value of 0.183 for the first operator and 0.632 for the second operator. In this case, low values suggest adequate reproducibility or consistency in the measurements made by each operator, i.e., the measurements can be repeated consistently, even when performed by different operators.

Furthermore, Student’s *t*-test for paired samples obtained *p*-values higher than 0.05 (0.375 for operator 1 and 0.330 for operator 2), meaning that there were no significant differences between the measurement values for either of the operators (Table 1).

The inter-operator error between operators 1 and 2 was 0.883, and the Student’s *t*-test for paired samples obtained a *p*-value of 0.074 > 0.05. Based on the results of this test, it was concluded that there were no statistically significant differences between the measurements made by operators 1 and 2. Although there was an error between them (0.883), this error was not large enough to consider that the measurements differed significantly. The consistency between operators was supported by the non-significant *t*-test result (section B in Table 1).

When comparing the measurements taken with CBCT and micro-CT, the error between instruments was 1.0061. This was calculated by taking the averages of the measurements obtained with CBCT by both operators and comparing them with the micro-CT measurements. Student’s *t*-test for paired samples obtained a *p*-value of 0.520 > 0.05, meaning that no statistically significant differences were identified between CBCT and micro-CT pulp volume measurements (section C in Table 1).

According to the results of Student’s *t*-test, no statistically significant differences were found in the pulp volume measurements obtained by CBCT and micro-CT. This suggests that both methods are comparable in terms of measuring the pulp volume and that there is no significant difference in the measurements between the two methods evaluated.

## 4. Discussion

Extensive research has been conducted on the use of CBCT in evaluating the internal structures of the endodontic system. The literature reveals that CBCT is highly effective in providing detailed images of the root canals, detecting anatomical variations, and identifying pathologies that may not be visible with traditional radiography. It has been particularly noted for its ability to assess the presence of periapical lesions, the quality of root fillings, and the detection of root fractures. The non-invasive nature of CBCT allows for a comprehensive assessment without causing discomfort to the patient. As such, it has become an indispensable tool in endodontic diagnostics, enabling practitioners to make more informed decisions regarding treatment strategies [32].

The present study was designed to validate the use of CBCT for the measurement of the human pulp chamber volume using the upper central incisors as a model. We compared the results of CBCT images with those obtained with micro-CT, which is regarded as the gold standard in studying the pulp chamber. We chose the upper central incisors, as chosen previously by Porto et al. [29], since they are shorter than the canines, they have wider pulp chambers than the lower incisors, and their root anatomy is simpler than that of the molars and premolars. Furthermore, volumetric measurements in multirooted teeth are less precise. Our results demonstrate that CBCT is comparable to micro-CT in evaluating the dental pulp chamber when the evaluators have appropriate training. Moreover, we support the idea that micro-CT is crucial in studies that evaluate the precision of measurements obtained with CBCT [33]. Thus, it may be assumed that CBCT achieves high precision and reproducibility for the evaluation of the pulp camber volume ex vivo. Nevertheless, measurements made on CBCT images present some issues, and it was observed that CBCT generated larger measurements than micro-CT, and different values can be obtained with different CBCT devices [25,34]. To the best of our knowledge, this is the first study to compare both techniques in the evaluation of the pulp chamber volume. It was used earlier to evaluate root canals [6,10].

Knowledge of the anatomy and dimensions of the root canals and pulp chamber is of interest in several branches of dentistry. First, for diagnostics and treatment in the field of endodontics [35,36], it facilitates operative sequences about choosing the most appropriate type of obturation or in guided root canal treatment [37]. Furthermore, CBCT is also useful to estimate age by calculating pulp chamber volumes, since there is an age-related reduction in the pulp chamber due to the deposition of secondary dentin [17]. The measurement of the pulp volume is of particular interest as it can be used to estimate the chronological age of a living or deceased human [38]. Various methods of determining the age of an individual are available depending on the subject’s age group. In individuals aged up to 24 years, age can be estimated based on dental eruption and the extent of dental development. However, once third molar maturation has taken place in adulthood, this becomes much more complex [16]. One of the characteristics that can be investigated to determine age is the decreasing size of the pulp cavity. Secondary dentin is deposited on the pulp cavity walls throughout an individual’s life, reducing its size; so, the pulp volume is indicative of age in adults [30].

The precise knowledge of the pulp chamber volume is also a key factor in preparing 3D scaffolds for tissue engineering for the regeneration of the dental pulp [18,19,20,39]. However, another important application of CBCT-generated images in dentistry is in the preclinical teaching and training of endodontic postgraduates. A need for realistic tooth models for education has often been expressed by dental students. Thus, 3D-printed replicas of teeth have been proposed for the creation of realistic macro-models to study anatomical details for use in preclinical dental education, as alternatives to the study of extracted human teeth [23,40]. This 3D printing technology offers new possibilities for dental schools, allowing them to create their own customized teaching models according to the specific curricula [41]. On the other hand, 3D models are optimal to study in detail the root canal anatomy and the ideal access cavity, and therefore for endodontic education during preclinical courses both before and during training [21,22,24,41]. Thus, based on the present results of CBCT, models of the different teeth could be created to study the morphology and dimensions of the dental cavity for educational and training purposes.

The objective of this study was to compare pulp volume measurements obtained by means of CBCT and micro-CT to confirm the precision of cone-beam computed tomography in measuring this variable. The results did not present statistically significant differences between the two measurement techniques, so it may be assumed that CBCT achieves high precision and reproducibility when calculating the pulp volume ex vivo. To the best of our knowledge, the literature does not include any other study that has made this comparison.

Micro-CT was the reference standard for the study. Its precision when studying small volume structures, such as reduced pulp chambers and mineralized pulp tissue, has already been satisfactorily assessed in the literature [31]. Currently, the use of micro-CT is considered crucial in studies that evaluate the precision of measurements obtained with CBCT [33].

Although recent studies have recommended the use of CBCT to assess root canals [6,10], the literature also describes imprecision in its measurements. Among the researchers who have set out to validate CBCT as a measurement tool, Celikten et al. studied volumetric distortion in 30 endodontically treated lower incisors, comparing measurements taken from micro-CT and CBCT with a voxel size of 200 microns. It was concluded that there were differences between the two sets of measurements, whereby CBCT generated larger measurements. Differences were also found between different types of CBCT, with greater variations found with the Promax 3D Max (Planmeca Olan Inc, Roselle, IL, USA) than the NewTom VGi evo (NewTom, Verona, Italy) [25]. This could be due to the presence of intracanal high-density materials, which could decrease the quality of the image. This was observed in the study by Møller et al. when comparing CBCT and micro-CT images in the search for voids within guttapercha-filled canals. False positives were found in the CBCT images, so the authors did not recommend CBCT for the assessment of the quality of the obturation of root canals [34]. In the present work, the root canals analyzed were intact, thus reducing the number of artifacts that could affect the precision of the measurements.

Our study had some limitations, including the size of the sample, although it was similar to the studies of Grande et al. [27] or Domark et al. [7]. Furthermore, our study was performed ex vivo, which could make CBCT’s volumetric measurements more imprecise due to superimposed structures [8]. It must be added that measurements were only taken with one type of CBCT device, the Promax 3D Max CBCT unit, and that the results could be different when using other brands. Puleio et al. also employed CBCT scanners from the same manufacturer when determining the volume of the root canal and gas bubbles in the investigation of the vapor lock phenomenon [28]. The voxel size is another parameter that is modifiable and influences the measurement accuracy [30,42]. Maret et al. (2012) scanned 70 teeth, taking volume measurements by means of CBCT set at different voxel sizes (200, 300, and 74 microns) and micro-CT (at 41 microns). It was found that with a voxel size of 300 microns, the measurements were significantly smaller [30]. In 2014, Maret et al. also compared geometric measurements between 37 dental reconstructions using CBCT with different voxel sizes and micro-CT. Images were placed on the same plane, and differences were identified using a color map; it was found that the maximum differences occurred at the cervical margins, cusp tips, and incisal edges. It was concluded that a voxel size of 200 microns or smaller should be used to study the dental anatomy [42]. This was the size used in the present study.

The study’s findings have significant implications for both clinical practice and research. Clinically, CBCT’s precision mirrors that of micro-CT, making it a reliable tool for dental pulp chamber evaluation. This accuracy is crucial in enhancing endodontic diagnosis and treatment, potentially reducing complications and improving patient outcomes.

In the research domain, the study’s validation of CBCT paves the way for its broader application in dental research. Additionally, the study supports the integration of CBCT into dental education, underscoring its value as a teaching tool for both undergraduate and postgraduate students. Overall, the study reinforces CBCT’s indispensable role in advancing dentistry.

In conclusion, the Promax 3D Max CBCT unit has proven to be a precise and reproducible tool in the ex vivo measurement of the pulp volume of the root canals in non-endodontically treated incisors. Further studies are necessary using other brands of CBCT devices and other dental groups. Studies are in progress in our laboratory to definitively validate CBCT for volume measurements in different teeth and using different CBCT devices.

## 5. Conclusions

This study demonstrated that cone-beam computed tomography (CBCT) is a precise and reproducible tool for the ex vivo evaluation of the dental pulp chamber volume. The results showed no statistically significant differences between volume measurements obtained by CBCT and micro-CT. Additionally, it was found that the measurements could be consistently replicated, even when performed by different operators.

The significance of the study lies in the validation of CBCT to measure the pulp chamber volume, which has significant implications in various branches of dentistry. This includes diagnosis and treatment in endodontics, age estimation through pulp chamber volume calculation, or the preparation of 3D realistic dental models for teaching and preclinical training. Furthermore, this study lays the groundwork for future research utilizing different CBCT device brands and different dental groups.

An interesting fact that emerges from the present study is that dental students should be trained in the management of CBCT and the interpretation of images obtained with this method. The possibility of obtaining three-dimensional images with CBCT facilitates the acquisition of knowledge of the anatomy of the pulp chamber and consequently will facilitate clinical actions on it in the future.

## Figures and Tables

**Table 1:** Differences in operators’ measurements and comparison of measurements taken with CBCT and micro-CT.

	Paired Differences					
				95% Confidence Interval of the Difference		
	Mean	SD	SEM	Lower	Upper	*p*
**A. Differences in operator 1 and operator 2 measurements**						
*Observer 1*						
CBCT 1st						
read-CBCT	−0.200	1.215	0.222	−0.654	0.254	0.375
2nd read						
*Observer 2*						
CBCT 1st						
read-CBCT	−0.200	0.894	0.222	−0.619	0.219	0.074
2nd read						
**B. Differences in operator 1 and operator 2 measurements**						
*Observer 1*						
	−0.350	0.828	0.185	−0.737	0.037	0.074
*Observer 2*						
**C. Comparison of pulp volume measurements obtained from CBCT and micro-CT**						
CBCT						
	−0.108	0.674	0.164	−0.239	0.454	0.50
micro-CT						

S.D.: standard deviation; S.E.M.: standard error of the mean.

## Data Availability

The data that support the findings of this study are available from the corresponding author upon reasonable request.
